# Microporous Polysaccharide Hemosphere Absorbable Hemostat Combined With a Spray-Type Adhesion Barrier Enhances the Safety and Efficacy of Repeat Robotic Liver Resection: Initial Experience and Outcomes

**DOI:** 10.7759/cureus.104141

**Published:** 2026-02-23

**Authors:** Takahisa Fujikawa, Yusuke Uemoto, Kei Harada, Keiji Nagata

**Affiliations:** 1 Surgery, Kokura Memorial Hospital, Kitakyushu, JPN

**Keywords:** absorbable hemostat, adhesion barrier, microporous polysaccharide hemosphere, repeat liver resection, robotic liver resection

## Abstract

Background

The use of spray-type adhesion barrier (SAB) can reduce the adhesion formation after liver resection, and some types of absorbable hemostats are reported to have anti-adhesive properties. The safety of repeat hepatectomy remains unknown when microporous polysaccharide hemosphere (MPH) is used in combination with SAB during a previous hepatectomy.

Methods

This single-center retrospective study included a total of 47 consecutive cases that underwent robotic repeat hepatectomy (RRH) at our institution between September 2021 and October 2025. The included patients were divided into three groups: patients who underwent a previous hepatectomy without the use of SAB (non-SAB group, n=15), those with the previous use of SAB only (SAB group, n=21), and those with the previous use of both MPH and SAB (MPH+SAB group, n=11). The perioperative outcomes were compared between the groups.

Results

There were no differences between the groups in background characteristics, difficulty scores, intraoperative blood loss, console time, operative time, or the occurrence of postoperative complications. In the whole cohort, there was no conversion to open hepatectomy and no mortality. While no significant difference was observed between the groups for the duration of adhesiolysis prior to robotic intervention, there were significant differences between the non-SAB, SAB, and MPH+SAB groups in the adhesion severity score (7 vs 6 vs 3, p < 0.001) and the time for robotic adhesiolysis (97.4 min vs. 42.4 min vs. 32.6 min, p = 0.002). The post-hoc pairwise comparison showed that the adhesion severity was less severe in the MPH+SAB group than in the non-SAB group (p < 0.001) or SAB group (p = 0.026).

Conclusions

The combined use of MPH and SAB does not appear to correlate with a higher incidence of intraperitoneal abscess or bile leakage during RRH. Furthermore, their use in a prior hepatectomy may enhance the safety of subsequent RRH by reducing the adhesion severity around the liver. Their application may also help minimize the duration of robotic adhesiolysis surrounding the liver, especially around the previous liver cut surface. Thus, the combined use of MPH and SAB appears feasible and may not be associated with increased complications, but a larger prospective validation is warranted to produce more trustworthy recommendations.

## Introduction

With advances in aggressive multimodal treatment for advanced cancer, debate continues regarding the safety and oncological benefits of repeat hepatectomy for intrahepatic recurrence of hepatocellular carcinoma (HCC) and colorectal cancer liver metastases (CRCLM) [1,2]. Intraperitoneal adhesions may develop after initial hepatectomy, potentially affecting both the oncological cure rate and safety of repeat hepatectomy. Therefore, adhesions, especially around the resection plane or hepatoduodenal ligament (HDL), are a major technical factor affecting postoperative difficulty, the risk of postoperative revisions, and operative time. In recent years, robotic surgery has become widespread in multiple medical fields, and its advanced functional devices have made robotic hepatectomy a preferred option for repeat hepatectomy [3-5].

Among the available adhesion barrier agents, the spray-type adhesion barrier (SAB, AdSpray™, Terumo, Tokyo, Japan) exhibits gel-like properties derived from N-hydroxysuccinimide-enriched carboxymethyl dextrin polymer [6]. Extensive clinical experience has demonstrated its efficacy in patients undergoing open or robotic repeat liver resection [4,5,7], although SAB may be beneficial in general peritoneal fields but potentially problematic on raw hepatic transection surfaces. The prior study indicated that the solution state of the substance may hinder the necessary adhesion for healing at the liver's cut surface, potentially resulting in an increased incidence of intra-abdominal abscesses after open hepatectomy [5]. Consequently, the use of SAB should generally be avoided on the liver cut surface or the surrounding area.

Regarding the intraperitoneal adhesions after abdominal surgery, a number of topical hemostatic agents have been studied for their antiadhesive properties [8]. A study utilizing a rat peritoneal model indicated that microporous polysaccharide hemosphere (MPH) was one of the preferred agents in reducing adhesion formation [8]. We hypothesized that the combined use of MPH and SAB may not only be safely performed without increasing the incidence of bile leaks or intra-abdominal abscesses, but also reduce intraperitoneal adhesion formation at the liver cut surface or in the adjacent area. To date, there has been no thorough examination of the impact of topical hemostatic agents like MPH on adhesion-related complications in repeat hepatectomy, particularly in the context of robotic repeat hepatectomy (RRH).

The aim of the current study is to evaluate the safety and efficacy of the combined application of MPH and SAB in RRH.

## Materials and methods

**Figure 1:**
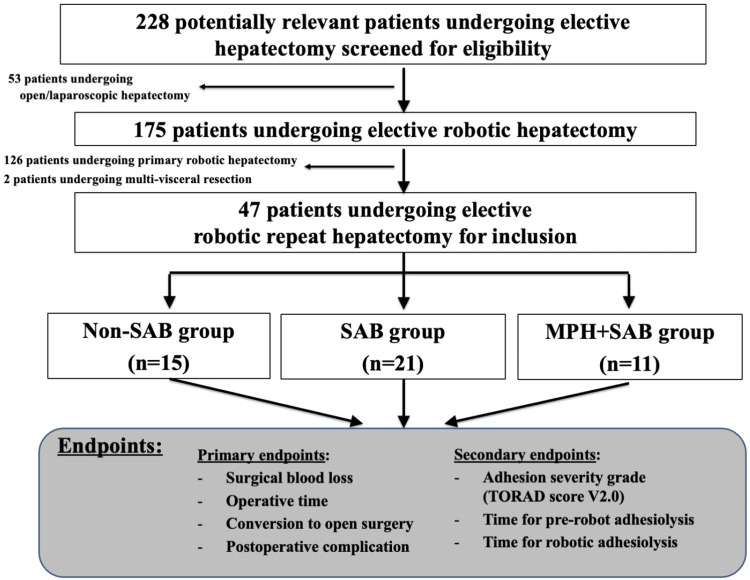
Consolidated Standards of Reporting Trials (CONSORT) diagram with the study design in the current study. SAB; spray-type adhesion barrier, MPH; microporous polysaccharide hemosphere, HDL; hepatoduodenal ligament.

For the current retrospective cohort study, we received approval from the institutional review board at Kokura Memorial Hospital (#25022751), and the single institution's prospectively gathered surgery database was checked for potentially pertinent instances. The exclusion criteria for robotic hepatectomy included patients with liver tumors measuring 10 cm or larger, as well as those with tumors deemed suitable for vascular reconstruction or multi-visceral resection. All other hepatectomy procedures, anatomical hepatectomy or hepatectomy for the posterosuperior regions, were planned to be conducted as robotic hepatectomy and included in the study. After excluding cases of primary robotic hepatectomy or those with synchronous surgery for other malignancies, we included a total of 47 consecutive RRHs, performed from September 2021 to October 2025, in the current study (Figure 1). Postoperative follow-up was scheduled three and six months after surgery with blood tests and CT scan imaging evaluation to assess delayed postoperative complications, including bile leakage.

To analyze the background and surgical factors of the whole cohort, the RRH patients were divided into three groups according to the application status of MPH and SAB: patients who underwent previous hepatectomy without the use of SAB (non-SAB group, n=15), those with the previous use of only SAB (SAB group, n=21), and those with the previous use of both MPH and SAB (MPH+SAB group, n=11). Whether or not SAB and/or MPH were applied after primary liver resection depended solely on the individual preference of the performing surgeon. The primary endpoints included surgical blood loss, operative time, conversion to open surgery, and postoperative complications; the secondary endpoints included the adhesion severity grade and time for adhesiolysis procedure (Figure 1).

The IWATE criteria were employed on a scale from 0 to 12 to evaluate the difficulty level of robotic liver resection [9]. The Clavien-Dindo classification (CDC) was utilized to categorize and evaluate postoperative complications, with complications classified as CDC class 3a or higher deemed significantly important [10]. Operative mortality refers to the incidence of death occurring within 30 days following a surgical procedure. To evaluate adhesion severity for patients in each group, we adopted the TORAD score V2.0, which grades adhesion severity based on the technical difficulty of repeat hepatectomy [11]. The TORAD score V2.0 was cumulative (score 0-9) on the basis of three adhesion conditions: the severity of adhesions around the hepatic hilum (score 0-3), those around the liver (score 0-3), and those at the site of the previous liver cut surface (score 0-3). Concerning the time for adhesiolysis, two distinct time intervals were predefined for analysis: (1) time for pre-robot adhesiolysis, the time interval defined from the beginning of laparoscopic adhesiolysis until the whole robotic ports were placed; and (2) time for robotic adhesiolysis, the time interval defined from the beginning of robotic adhesiolysis until the complete adhesiolysis around the scheduled liver resection area was performed.

Statistical analysis

Continuous variables were represented as median (range), whereas categorical data were displayed as absolute numbers and percentages. Fisher's exact probability test was employed for univariate comparisons of categorical values, while continuous variables for non-normally distributed data were assessed using Kruskal-Wallis tests, and the Bonferroni post-hoc test was used to determine pairwise differences. All P values were two-tailed, and P values below .05 were deemed statistically significant. All statistical analyses were conducted using EZR (Saitama Medical Center, Jichi Medical University, Shimotsuke, Japan), a graphical user interface for R version 2.13.0 (The R Foundation for Statistical Computing, Vienna, Austria) [12].

Surgical technique

No significant alterations occurred in the surgical procedures or protocols throughout the study period. All RRHs in the current cohort were performed using the Da Vinci Xi surgical system (Intuitive Surgical, Inc., Sunnyvale, CA, USA). The patient's position and port placement during RRH have been previously described [13,14]. Briefly, in the case of the anterolateral (S2, S3, S4b, S5, and S6) or superior segments (S8 and S4a), the patient was positioned supine with their legs spread apart in a 10-degree reverse Trendelenburg position. Five trocars were usually used for the procedure: the ports were placed concentrically around the targeted liver lobe, including four trocars (#1 to #4) for robotics at the umbilical level and one 12-mm trocar from the left-upper side for the assistant surgeon. On the other hand, in the case of the posterior segment, the patient was placed in a left lateral decubitus position, and the head side was raised 10 degrees. Robotic port #1 was positioned on the right upper lateral side of the abdomen, #3 was positioned above the umbilicus, #2 and a 12-mm assistance port were placed between ports #1 and #3, and #4 was positioned on the left-sided epigastrium.

Before liver parenchymal transection, the Pringle maneuver was typically prepared via the extraperitoneal tourniquet system as described in the preceding study [15] and implemented as necessary. Monopolar curved scissors or the double bipolar technique were commonly utilized for adhesiolysis or dissection around the liver. The saline-linked coagulation (SLiC) method, employing monopolar curved scissors or Maryland bipolar forceps with saline, was exclusively utilized for parenchymal transection [13,14]. In the case of only SAB use (the SAB group), SAB (AdSpray™, packages of 9.4 mL) was routinely applied widely around the HDL and liver surface, except for the site close to the liver cut surface. In the case of combined use of MPH and SAB (the MPH+SAB group), MPH (Arista™ Absorbable Hemostat, packages of 3 g, Becton, Dickinson and Company, Franklin Lakes, NJ, USA) was first applied to the liver resection surface. Subsequently, SAB was widely applied around the HDL and liver surface, including the area adjacent to the resection surface. In both cases, the application of MPH and/or SAB was performed just prior to the peritoneal closure.

Typical cases of RRH following a previous hepatectomy without the previous use of MPH are shown in Video 1. The first case was an S7 partial resection after a previous open left lobectomy without the previous use of MPH or SAB application. Strong adhesions were observed around the liver, especially at the previous resection surface, making dissection extremely difficult. Taping of the HDL was attempted, but due to strong adhesions on the posterior side of the HDL, the HDL was damaged, resulting in bleeding, and the Pringle maneuver was abandoned. Another case was an S2 partial resection after a previous S2 partial resection with the previous application of SAB but not MPH. The previous resection site had strong adhesions accompanied by neovascularization, making it bleed easily, and the dissection of this area took a considerable amount of time. These cases highlighted the need for measures to reduce adhesions around the liver cut surface, with a view to repeat hepatectomy.

**Video 1:** Cases of robotic repeat hepatectomy following previous hepatectomy without the use of MPH. MPH; microporous polysaccharide hemosphere.

**Figure 2:**
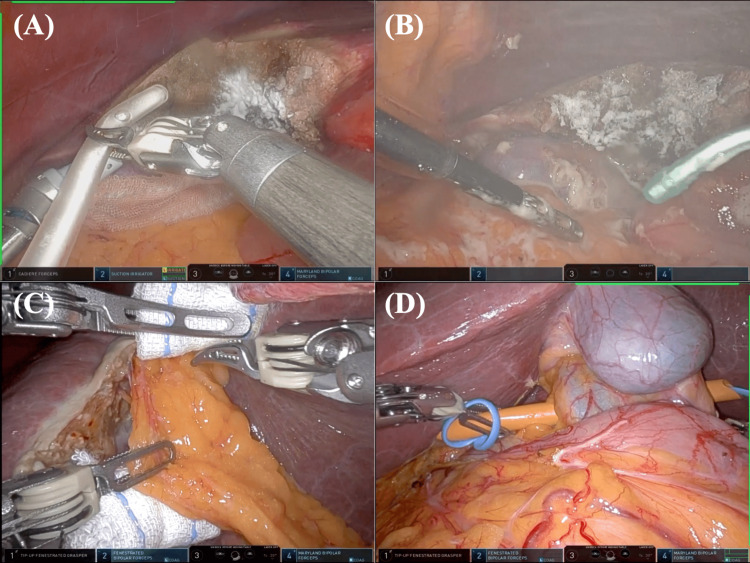
A robotic repeat hepatectomy case following S4b anatomical hepatectomy with the use of MPH combined with SAB. (A) In the final step of the previous robotic S4b anatomical hepatectomy, MPH was applied to the resected liver surface. (B) Subsequently, SAB was widely applied around the liver and HDL, including the area adjacent to the resection surface (Figure 1B). (C) During the following robotic S7 anatomical repeat hepatectomy, mild adhesion was limited to the previous liver cut surface, which was robotically detached within three minutes. (D) There was no adhesion around the HDL, and the successful taping of the HDL was performed. MPH; microporous polysaccharide hemosphere, SAB; spray-type adhesion barrier, HDL; hepatoduodenal ligament.

On the other hand, a typical case of RRH with the previous use of MPH combined with the SAB application is shown in Figure 2 and Video 2. In the final step of the previous robotic S4b anatomical hepatectomy, MPH was applied to the resected liver surface (Figure 2A). Subsequently, SAB was widely applied around the liver and HDL, including the area adjacent to the resection surface (Figure 2B). During the following RRH (S7 subsectionectomy of the liver), adhesion severity was low (TORAD score 1), and its area was limited to the previous liver cut surface, which was robotically detached relatively easily (Figure 2C). There was no adhesion around the HDL, and the successful taping of HDL was performed (Figure 2D). The scheduled robotic S7 subsectionectomy could be performed without any intraoperative events. In the final step of the procedure, a combined MPH and SAB application was performed.

**Video 2:** A robotic repeat hepatectomy case following S4b anatomical hepatectomy with the use of MPH combined with SAB. MPH; microporous polysaccharide hemosphere, SAB; spray-type adhesion barrier.

**Figure 3:**
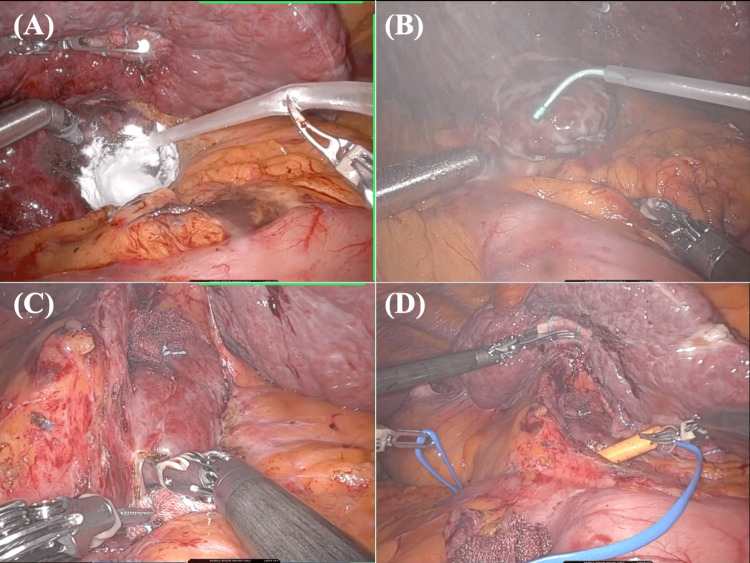
A robotic repeat hepatectomy case following S1 non-anatomical hepatectomy with the use of MPH combined with SAB. (A) In the final step of the previous robotic S1 non-anatomical hepatectomy, the MPH application to the liver cut surface was performed. (B) SAB was subsequently applied around the HDL and the area adjacent to the resection surface. (C) During the following RRH (S8 non-anatomical hepatectomy), adhesion was present around the HDL but limited to only the surface area. (D) The HDL could be successfully taped without any vessel injury or bleeding. MPH; microporous polysaccharide hemosphere, SAB; spray-type adhesion barrier, HDL; hepatoduodenal ligament, RRH; robotic repeat hepatectomy.

Another case of RRH, with the previous use of the MPH application combined with the SAB application, is shown in Figure 3 and Video 3. In the final step of the previous robotic S1 non-anatomical hepatectomy, the MPH application combined with SAB administration was performed (Figures 3A, 3B). SAB was applied not only around the liver but also at the area adjacent to the liver resection surface. During the following RRH (S8 non-anatomical hepatectomy), adhesion was present around the HDL but limited to only the surface area (TORAD score 4), and the successful HDL taping could be performed without any vessel injury or bleeding (Figure 3D). The scheduled robotic S8 non-anatomical hepatectomy could be performed without any intraoperative events. In the final step of the procedure, a combined MPH and SAB application was performed.

**Video 3:** A robotic repeat hepatectomy case following S1 non-anatomical hepatectomy with the use of MPH combined with SAB. MPH; microporous polysaccharide hemosphere, SAB; spray-type adhesion barrier.

## Results

Table 1 displays the patient and tumor characteristics in the current cohort. There were no differences in any background characteristics, including age, gender, the status of liver function, and the type of disease. The tumor characteristics, including the type of disease and size of the tumor, were also similar between the groups. Table 2 displays the surgical factors and outcomes for the included patients. No differences were found between the groups in the difficulty scores, intraoperative blood loss, console time, or operative time. In the whole cohort, there were no cases of grade B or C post-hepatectomy liver failure and no mortality. Only one postoperative complication with CDC 3a occurred (bile leak in one) in the SAB group. The patient received preoperative chemotherapy and subsequent robotic repeat S8 liver resection for recurrent CRCLM after the initial robotic S2 partial liver resection. He experienced postoperative bile leakage on day five, but it improved conservatively. Otherwise, there were no specific postoperative complications related to the use of MPH and SAB, such as intraperitoneal abscesses.

**Table 1:** Patient, laboratory, and tumor characteristics in the current cohort. ^&^Kruskal-Wallis test was used. ^$^Fisher’s exact probability test was used. Abbreviations: SAB, spray-type adhesion barrier; MPH, microporous polysaccharide hemosphere; BMI, body mass index; ICG-R15, indocyanine green retention test after 15 min; HCC, hepatocellular carcinoma; CRCLM, colorectal cancer liver metastasis.

Variables	Non-SAB (n=15)	SAB (n=21)	MPH+SAB (n=11)	P value
Age, y, median (range)	75 (66-85)	74 (49-86)	80 (43-89)	0.648^&^
Male gender, n (%)	11 (73.3)	14 (66.7)	7 (63.6)	0.857^$^
ICG-R15, %, median (range)	13.5 (4.8-36.8)	15 (3.3-29.5)	17.5 (5.8-31.2)	0.453^&^
Disease: HCC, n (%)	10 (66.7)	11 (52.4)	5 (45.5)	0.525^$^
Disease: CRCLM, n (%)	4 (26.7)	8 (38.1)	4 (36.4)	0.778^$^
Number of the tumor, median (range)	1 (1-3)	1 (1-3)	1 (1-2)	0.561^&^
Maximum size of the tumor, mm, median (range)	20 (10-32)	25 (15-33)	21 (10-28)	0.191^&^

**Table 2:** Surgical factors and outcomes in the current cohort. ^&^Kruskal-Wallis test was used. ^$^Fisher’s exact probability test was used. Abbreviations: SAB, spray-type adhesion barrier; MPH, microporous polysaccharide hemosphere; PSS, postero-superior segments; PHLF, post-hepatectomy liver failure; CDC, Clavien-Dindo classification; LOS, length of postoperative stay.

Variables	Non-SAB (n=15)	SAB (n=21)	MPH+SAB (n=11)	P value
No. of hepatectomy performed, median (range)	2 (2-5)	2 (2-4)	2 (2-6)	0.661^&^
Anatomical hepatectomy, n (%)	1 (6.7)	7 (33.3)	2 (18.2)	0.091^$^
Resection of PSS area, n (%)	9 (60.0)	6 (28.6)	4 (36.4)	0.158^$^
Difficulty score, median (range)	5 (1-10)	5 (1-10)	4 (2-9)	0.870^&^
Operative time, min, median (range)	298 (179-622)	273 (90-535)	264 (173-475)	0.541^&^
Console time, min, median (range)	180 (83-476)	194 (38-413)	182 (54-385)	0.630^&^
Blood loss, mL, median (range)	0 (0-60)	14 (0-60)	0 (0-81)	0.757^&^
Red blood cell transfusion, n (%)	0 (0.0)	0 (0.0)	0 (0.0)	NA
Conversion to open surgery, n (%)	0 (0.0)	0 (0.0)	0 (0.0)	NA
PHLF (Grade B, C), n (%)	0 (0.0)	0 (0.0)	0 (0.0)	NA
Operative mortality, n (%)	0 (0.0)	0 (0.0)	0 (0.0)	NA
Any postoperative complication (CDC 1 or higher), n (%)	1 (6.7)	2 (9.5)	1 (9.1)	0.952^$^
Severe postoperative complication (CDC 3a or higher), n (%)	0 (0.0)	1 (4.8)	0 (0.0)	0.614^$^
LOS, d, median (range)	8 (5-14)	8 (5-25)	10 (8-15)	0.488^&^

Figure 3 demonstrates the comparison of the adhesion severity score and the time for pre-robot and robotic adhesiolysis between the groups. As shown in Figure 3A, there were significant differences in the adhesion severity score between the non-SAB, SAB, and MPH+SAB groups (TORAD score 7 vs. 6 vs. 3, p < 0.001), and the post-hoc pairwise comparison showed that the score was significantly lower in the MPH+SAB group than in the non-SAB group (3 vs. 7, p < 0.001) or in the SAB group (3 vs. 6, p = 0.025). No significant difference was observed between the groups in the duration of adhesiolysis prior to robotic intervention (Figure 3B, non-SAB 58 min vs. SAB 49 min vs. MPH+SAB 58 min, p = 0.428). Although the time taken for robotic adhesiolysis was markedly reduced in the MPH+SAB and SAB groups compared to the non-SAB group (Figure 3C, 28 min vs. 41 min vs. 75 min, p = 0.013), the post-hoc analysis showed no significant differences between the MPH+SAB and SAB groups (p = 1.000).

**Figure 4:**
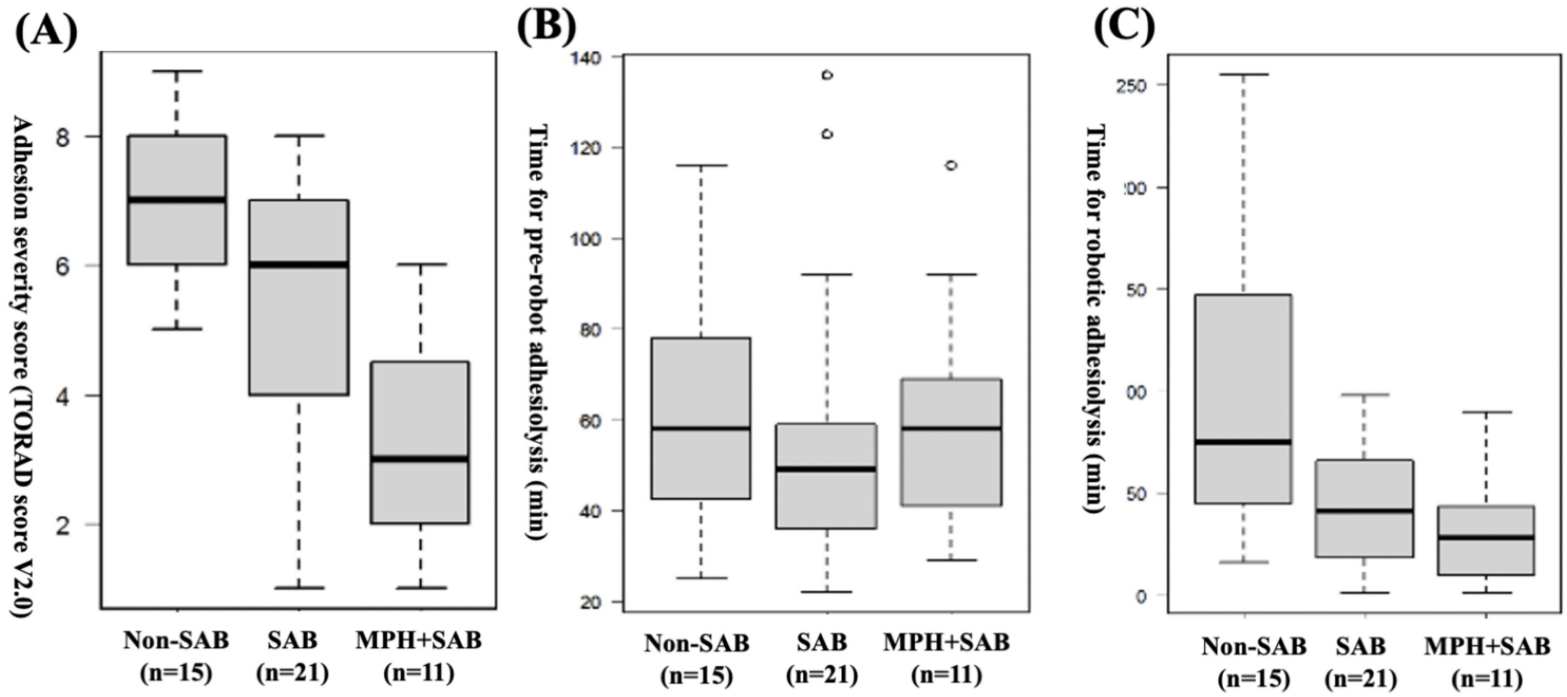
Comparison of the adhesion severity score and the time for pre-robot and robotic adhesiolysis between the groups. (A) There were significant differences in the adhesion severity score between the non-SAB, SAB, and MPH+SAB groups (TORAD score 7 vs. 6 vs. 3, p < 0.001), and the post-hoc pairwise comparison showed that the score was significantly lower in the MPH+SAB group than in the SAB group (3 vs. 7, p < 0.001) or in the SAB group (3 vs. 6, p = 0.025). (B) No difference was found between the groups in the time required for adhesiolysis before robotic operation (non-SAB 58 min vs. SAB 49 min vs. MPH+SAB 58 min, p = 0.428). (C) The time for robotic adhesiolysis was significantly reduced in the MPH+SAB and SAB groups compared to the non-SAB group (28 min vs. 41 min vs. 75 min, p = 0.013). The post-hoc analysis showed no significant differences between the MPH+SAB and SAB groups (p = 1.000). (A, B, C) Kruskal-Wallis test was used. MPH; microporous polysaccharide hemosphere, SAB; spray-type adhesion barrier.

## Discussion

In the current single-center retrospective cohort study, we evaluated the safety and efficacy of the combined application of MPH and SAB in RRH. In the whole RRH cohort, there was no conversion to open hepatectomy, no mortality, and no increase in intra-abdominal abscess or biliary leakage. Although the background characteristics, difficulty scores, operative and console time, and surgical blood loss were similar between the groups, the less severe adhesion and the shorter time required for robotic adhesiolysis were achieved in the MPH+SAB and SAB groups. Post-hoc pairwise comparison demonstrated a lower adhesion severity score in the MPH+SAB group compared to the SAB group. Thus, the combined use of MPH and SAB in RRH appears feasible and may not be associated with increased complications.

Arista™ Absorbable Hemostat, a commercially available MPH powder, is derived from a biodegradable plant source, fully absorbs within 24 to 48 hours, and has no animal or human components; hence, it is deemed physiologically safe. MPH augments intrinsic coagulation processes by sequestering water and low molecular weight substances from the blood, so concentrating platelets and clotting proteins on its beaded surface. It has been utilized in surgical procedures and has demonstrated efficacy in reducing intraoperative and postoperative hemorrhaging [16-18]. Concerning liver resection, certain publications indicate the safety and efficacy of MPH in both open and robotic liver surgery [18,19].

In addition to the hemostatic property, several hemostatic agents, including MPH, are reported to have anti-adhesive properties during abdominal surgery [8,20,21]. The plausible pathways for anti-adhesive effects of hemostatic agents might be considered as follows: once improved hemostasis is achieved, hematoma and/or inflammation at the local site might be minimized, and bile contamination could be avoided, resulting in a barrier-like effect during the immediate critical period of adhesion formation. A study utilizing a rat peritoneal model indicated that MPH effectively reduced adhesion formation. Its application to the peritoneum and subsequent observation after seven days revealed no residual agent present and no significant difference in acute inflammation compared to controls [8]. Based on the positive research findings and the functionally safe properties of MPH, including its rapid absorption, we currently favor the use of MPH not only to promote hemostasis but also to minimize adhesions during minimally invasive hepatectomy. The present study demonstrated that the combined use of MPH and SAB was safely applied without an increase in bile leakage or intra-abdominal abscess, and also their use might reduce the adhesion severity, especially in the area adjacent to the previous liver cut surface.

Following extensive experience in a clinical setting, the safety and efficacy of SAB have been reported in patients undergoing open or RRH [4,5,7]. Since hepatectomy can cause unique postoperative complications associated with hepatic parenchymal resection, such as bile leakage and intractable ascites [22,23], the application of SAB should be generally avoided on the liver cut surface or the area adjacent to the cut surface [5]. Currently, during a robotic hepatectomy, we routinely use MPH combined with SAB in order not only to promote hemostasis but also to reduce adhesions, especially in the area adjacent to the liver cut surface. In the final phase of liver parenchymal transection, MPH is first applied to the liver cut surface, followed by a broad application of SAB around the liver and HDL, encompassing the region adjacent to the liver cut surface. The present study demonstrated that the combined application of MPH and SAB during RRH may not be associated with an increase in the incidence of postoperative complications, and also, its previous application may reduce the severity of adhesion around the liver, especially at the site of a previous liver cut surface, during RRH.

Limitations of the study

This study has several limitations. First, because of the study’s non-randomized cohort design, patient selection may be subject to bias and potential risk of conflicts of interest. Second, the study's applicability in determining the impact of treatments on outcomes is limited by its retrospective design. Third, the sample size was limited; an increased sample size would likely produce more reliable recommendations. Fourth, the current study lacks long-term oncological follow-up. Nevertheless, this approach might represent a significant advancement toward the standardization of RRH. This method may enhance security and outcomes, representing a further advancement toward the safe and effective general application of MPH and SAB in robotic hepatectomy.

## Conclusions

The combined use of MPH and SAB may not be associated with an increase in the incidence of postoperative complications, including bile leakage or intraperitoneal abscess during RRH. In addition, their application during the previous hepatectomy can facilitate a secure RRH with reduced severity of adhesion around the liver. Their application also leads to minimizing the duration of robotic adhesiolysis surrounding the liver, especially around the previous liver cut surface. Thus, their combined use in RRH appears feasible and may not be associated with increased complications, but the larger prospective validation is warranted to produce more trustworthy recommendations.
